# Clostridium difficile Infection in Liver Cirrhosis Carries a Higher Risk of Mortality: A Comprehensive Literature Review

**DOI:** 10.7759/cureus.5463

**Published:** 2019-08-22

**Authors:** Veeraraghavan Meyyur Aravamudan, Shahab R Khan, Ikram Hussain

**Affiliations:** 1 Internal Medicine, Woodlands Health Campus, Singapore, SGP; 2 Internal Medicine, Banner University Medical Center, University of Arizona, Tucson, USA; 3 Internal Medicine: Gastroenterology, Woodlands Health Campus, Singapore, SGP

**Keywords:** clostridium difficile infection, cirrhosis, mortality

## Abstract

Clostridium difficile (C. difficile) infection is associated with higher mortality in liver cirrhosis. This literature review discusses the risk factors associated with increased mortality in patients with C. difficile infection in liver cirrhosis. This literature review also highlights the importance of selecting antibiotics wisely, carefully selecting patients who are candidates for antibiotic prophylaxis for spontaneous bacterial peritonitis in liver cirrhosis and avoiding unnecessary proton pump inhibitors in liver cirrhosis.

## Introduction and background

Introduction

The presence of antimicrobials is a very strong risk factor for Clostridium difficile (C. difficile) infection (CDI) because of their perturbation of the intestinal microbiome. Patients with cirrhosis are frequently administered chronic antimicrobial therapies, such as prophylaxis, for spontaneous bacterial peritonitis, which puts them at a higher risk of CDI. Patients with CDI and cirrhosis have worse outcomes as compared with patients who have cirrhosis and diarrhea but not a C. difficile infection.

Burden on healthcare

CDI was found to be an independent risk factor for mortality in hospitalized liver disease patients, emphasizing the importance of having a high index of suspicion for the early diagnosis and appropriate initiation of treatment. There is also a difficulty in discharging these cohorts of patients, which unfortunately can increase the length of stay in a healthcare facility.

Objectives

The objectives of this literature review is to identify the risk factors associated with increased mortality in patients with CDI in liver cirrhosis.

Pathology

Patients with cirrhosis may be at particular risk of developing CDI for three reasons. First, antibiotic use is common in cirrhosis patients. Prophylactic use of broad-spectrum quinolone or beta-lactam antibiotics is standard practice to prevent infections and reduce mortality in cirrhotic patients [1]. Second, cirrhotic patients also commonly receive proton pump inhibitor (PPI), both for established indications, such as symptomatic gastroesophageal reflux, and prior peptic ulcer disease, as well as for unproven indications such as healing of esophageal ulcers after endoscopic band ligation [1]. Finally, there is frequently a need for hospitalization to treat complications of cirrhosis, such as variceal bleeding, ascites, or encephalopathy, which places patients in an environment in which there is a high likelihood of exposure to C. difficile [1]. Patients with cirrhosis have an impaired local gut immune response, increased bowel wall edema, and poor intestinal motility, all of which can promote perturbations of the intestinal microflora and bacterial overgrowth [1].

## Review

Materials and methods

We conducted a literature search of journal articles using the US National Library of Medicine PubMed database, PubMed, MEDLINE, Embase, Cochrane Library, and Google Scholar databases, ClinicalTrials.gov for studies, and ISI Web of Science. No date restrictions were placed on the search. A thorough search for controlled clinical trials and cohort studies was conducted. We used the keywords "clostridium difficile infection" and "cirrhotic liver disease."

Included studies were studies published in English that assessed the association between CDI and cirrhotic liver disease. Reference lists were also screened. From the search results, articles with irrelevant titles were discounted, with the remaining abstracts examined for relevance.

The authors of this review independently determined the eligibility of studies and assessed the methodology of the included studies. In this review article, we will discuss the risk factors associated with increased mortality in patients with CDI in liver cirrhosis. See Table 1 below for more on risk factors for patients with CDI.

**Table 1:** Review of studies on Clostridium difficile infection in liver cirrhosis CDAD: C. difficile-associated diarrhea; CDI: Clostridium difficile (C. difficile) infection; LOS: length of stay; PPI: proton pump inhibitor; WBC: white blood cell; HA-CDI: hospital-acquired-CDI; MELD: model end-stage liver disease

Study Author(s)	Study Name	Findings
Pepin et al. [2]	Clostridium difficile-associated diarrhea in a region of Quebec from 1991 to 2003: A changing pattern of disease severity.	Predictors of severe disease in patients with CDAD include age over 65, fever, nosocomial acquisition, nasogastric tube placement, immunosuppression, peak WBC, and peak creatinine.
Kruger et al. [3]	Early readmission predicts increased mortality in cirrhosis patients after Clostridium difficile infection.	Patients with CDI and cirrhosis experienced higher 30-day readmission rates (33% vs. 24%), index admission mortality (5% vs. 2.5%), and calendar-year mortality (9% vs. 4%) than CDI patients without cirrhosis. Further, recurrent CDI and cirrhosis-related complications were two of the biggest causes of readmission.
Bajaj et al. [1]	Clostridium difficile Is associated with poor outcomes in patients with cirrhosis: A national and tertiary center perspective.	Patients with CDAD and cirrhosis experienced higher rates of mortality (13.8% vs. 8.2%), LOS (14.4 days vs. 6.7 days), and charges ($79,351 vs. $35,686) than patients with cirrhosis but not CDAD, and PPI use was significantly higher in patients with cirrhosis and CDI than those with cirrhosis and no CDI (40 / 54, 74% vs. 38 / 108, 35%).
Soica et al. [4]	Clostridium difficile infection in hospitalized cirrhotic patients with hepatic encephalopathy.	About 7% (17 out of 231) of cirrhotic patients admitted with hepatic encephalopathy were infected with C. difficile, and rifaximin was used in 219 of these patients. About 8% of cirrhotic patients developed diarrhea when treated with rifaximin, although none were diagnosed with CDI.
Bajaj et al. [5]	Second infections independently increase mortality in hospitalized patients with cirrhosis: The North American Consortium for the Study of End-stage Liver Disease (NACSELD) experience	Out of 207 patients hospitalized with cirrhosis, 10 were infected with C. difficile, 6 acquired it during their second hospitalization, and the case fatality rate for the second hospitalization was higher than those with cirrhosis of the liver (40%).
Smith, Northup, Argo [6]	Predictors of mortality in cirrhosis inpatients with Clostridium difficile infection.	The study found that hypoalbuminemia and admission to the ICU are strong predictors of increased mortality rate in patients with cirrhosis and C. difficile infection.
Ali et al. [7]	Clostridium difficile infection in hospitalized liver transplant patients: A nationwide analysis.	Cirrhotic patients with a liver transplantation discharge are more likely to have CDI (2.7%) than non-liver transplant cirrhotic patients (0.9%).
Banks et al. [8]	Trends in mortality following Clostridium difficile infection in Scotland, 2010–2016: A retrospective cohort and case-control study.	The results of this study suggested that cirrhotic patients with CDI are subject to an almost 300% increase in 30-day mortality; in addition, age, a high Charlson score, HA-CDI, and liver, heart, and malignancy comorbidities are correlated with higher mortality rates.
Sundaram et al. [9]	Effects of Clostridium difficile Infection in patients with alcoholic hepatitis.	Patients with alcoholic hepatitis who also have CDI are at greater risk for inpatient mortality than alcoholic hepatitis patients without CDI.
Dotson et al. [10]	Outcomes associated with Clostridium difficile infection in patients with chronic liver disease.	This study found that patients with CDI were more prone to in-hospital mortality (8.8% vs. 18.6%), had prolonged hospital stays (1.19 more days), and were subject to an average of $8,632 more in total costs.
Hong, Feuerstadt, & Brandt [11]	MELD is the only predictor of short-term mortality in cirrhotic patients with C. difficile infection.	Cirrhotic patients with CDI have higher 30-day mortality rates than cirrhotic patients without CDI, and MELD is a significant contributor to short-term mortality.
Rosenblatt et al. [12]	The rise of Clostridioides difficile infections and fall of associated mortality in hospitalized advanced cirrhotics.	Researchers discovered that advanced cirrhotic patients with CDI experienced higher mortality rates (OR 1.47) and were more likely to have acute kidney injury (OR 2.09).
Saab et al. [13]	Hospitalized patients with cirrhosis should be screened for Clostridium difficile colitis.	This study found that screening cirrhotic patients for CDI, and subsequently treating them if positive, resulted in significant savings on healthcare costs and a significant decrease in care required.

Results of the review

Risk factors for a poor outcome in patients with C. difficile-associated diarrhea (CDAD) previously identified in published studies included impaired cognitive status, functional incapacity, recent endoscopy, age ≥ 65, elevated peak white blood cell (WBC), elevated peak creatinine, and immunosuppression [2]. Immunosuppression has previously been defined as a group including patients with human immunodeficiency virus (HIV), leukemia, lymphoma, neutropenia, organ transplant recipients, and systemic glucocorticoid use and liver cirrhosis. The findings of the literature review are discussed in the table above, but we will discuss the findings of the studies which ascertains the relationship between Clostridium difficile and liver cirrhosis.

The results of Bajaj et al. showed that patients with CDAD and cirrhosis experienced higher rates of mortality (13.8% vs. 8.2%), length of stay (LOS) (14.4 days vs. 6.7 days), and charges ($79,351 vs. $35,686) than patients with cirrhosis but not CDAD, and PPI use was significantly higher in patients with cirrhosis and CDI than those with cirrhosis and no CDI (40/54, 74% vs. 38/108, 35%) [1]. Patients with cirrhosis are frequently prescribed prophylactic antibiotics for spontaneous bacterial peritonitis and proton pump inhibitors for prophylaxis of gastrointestinal bleeding. When admitted, cirrhotic patients received antibiotics more often, which put them at higher risk of C. difficile.

Kruger and colleagues showed that cirrhotic patients hospitalized for CDI had higher rates of readmission and mortality than those without cirrhosis [3]. Colectomy and decompensated cirrhosis predicted index admission mortality, whereas readmission within 30 days of index hospitalization and decompensation were predictors of calendar-year mortality. Approximately two-thirds of the cirrhosis cohort was decompensated; these patients had more readmissions, mortality, and health care resource utilization than those with compensated cirrhosis. Early readmission as a predictor of calendar-year mortality is a novel finding of Kruger’s study. Age, gender, and renal failure also predicted calendar-year mortality, but do not represent modifiable risk factors.

Bajaj et al. showed that second infections independently increase mortality in hospitalized patients with cirrhosis. Out of 207 patients hospitalized with cirrhosis, 10 were infected with C. difficile, six acquired it during their second hospitalization, and the case fatality rate for the second hospitalization was higher than those with cirrhosis of the liver (40%) [1]. In the study by Smith and colleagues, the authors found that hypoalbuminemia and admission to the ICU are strong predictors of increased mortality rate in patients with cirrhosis and C. difficile infection. Physicians should consider these groups with hypoalbuminemia and intensive care unit (ICU) admission as high risk and consider starting empirical vancomycin. Again, these are small studies and bigger clinical trials and more research are required to prove this point. Banks et al. found that cirrhotic patients with CDI are subject to an almost 300% increase in 30-day mortality; in addition, age, a high Charlson score, hospital-acquired-CDI (HA-CDI), and liver, heart, and malignancy comorbidities are correlated with higher mortality rates.

Sundaram et al. found that alcoholic hepatitis patients who also have CDI are at greater risk for inpatient mortality than alcoholic hepatitis patients without CDI [9]. Hong et al. found that cirrhotic patients with CDI have higher 30-day mortality rates than cirrhotic patients without CDI, and model end-stage liver disease (MELD) is a significant contributor to short-term mortality [11]. Rosenblatt et al. found that CDI independently increased mortality in advanced cirrhotics (OR 1.47, P < 0.001) and was associated with acute kidney injury (AKI) (OR 2.09, P < 0.001), which itself significantly increased mortality (OR 4.54, P < 0.001) [8].

Discussion

The study of the intestinal microbiome is an exciting area of current research that may provide additional knowledge on this important topic. Although little is known about the microbiome in liver disease, emerging data suggest that there may be a relationship between microbial composition and liver disease. The role of the microbiome as it relates to C. difficile colonization and disease is quite clear, and several studies have shown that prior hospitalization is a risk factor for colonization. Prior hospitalization for an infection or severe illness (with or without antibiotic therapy) may contribute to microbiome disruption, which in turn increases the probability that C. difficile will colonize. Furthermore, a study of the restoration of the microbiome with probiotics is challenged by the wide range of combinations and doses in probiotic usage, resulting in little clear evidence of the superiority of any one type. A growing body of evidence in this area is likely to emerge in the coming years with the ultimate goal of decreasing CDI-related morbidity and mortality among patients with cirrhosis and CDI have higher mortality.

Saab and colleagues have recommended that screening patients with cirrhosis on admission to the hospital, with isolation and treatment to eradicate C. difficile, will decrease mortality and healthcare costs [13]. There is a reluctance on the universal screening of C. difficile among clinicians and if every colonized C. difficile patient were treated with antibiotics, the excess risks of antibiotic resistance must also be carefully considered. Prevention and/or early diagnosis with more consistent eradication of the initial infection would prove beneficial. The use of lactulose and probiotics reducing the risk of CDI in cirrhotics still needs additional evidence. Although not specifically studied in patients with cirrhosis, strategies to decrease CDI in the general population should also be stringently applied to cirrhotics.

Strengths of the study

This is one of the most comprehensive literature reviews discussing the increased mortality of cirrhotic patients infected with C. difficile. This is also the first literature review assessing all the risk factors associated with increased mortality in cirrhotics infected with CDI. This study has been done in multiple centers, which will increase the generalizability of the results within a population.

Limitations

Most of the studies discussed in this literature review are retrospective and done with a small sample size. Testing for C. difficile toxin was done at the clinicians' suspicion and no standardized protocols were carried out for testing for C. difficile toxin. It is also difficult to assess the severity of illness and the classification of liver cirrhosis, whether early or advanced, although the model for end-stage liver disease (MELD) score can buffer the wide variation. Various aspects of interest such as race/ethnicity, medications, and outpatient care are not discussed in detail. We need more evidence to discuss the complex relationship between liver cirrhosis and C. difficile infection and good clinical studies with large sample size are needed to address our research question.

The study is limited by the use of different practice patterns over the different centers and by the lack of a control group without infections. Most of the studies are done in a tertiary referral center and the intensity of liver disease may be more severe.

## Conclusions

Cirrhotic patients are at a higher risk of C. difficile infection due to an underlying immunodeficiency and a higher frequency of hospitalizations. Early diagnosis and treatment with CDI in cirrhotic is of paramount importance in view of its association with mortality. Physicians should be aware that recurrent CDI in cirrhotic patients carries higher mortality. Prevention, early recognition, and aggressive treatment strategies need to be re-examined for those patients with cirrhosis and CDI to prevent deleterious consequences.
